# Application of palaeogenetic techniques to historic mollusc shells reveals phylogeographic structure in a New Zealand abalone

**DOI:** 10.1111/1755-0998.13696

**Published:** 2022-08-21

**Authors:** Kerry Walton, Lachie Scarsbrook, Kieren J. Mitchell, Alexander J. F. Verry, Bruce A. Marshall, Nicolas J. Rawlence, Hamish G. Spencer

**Affiliations:** ^1^ Otago Palaeogenetics Laboratory, Department of Zoology University of Otago Dunedin New Zealand; ^2^ Palaeogenomics and Bio‐Archaeology Research Network, School of Archaeology University of Oxford Oxford UK; ^3^ Centre for Anthropobiology and Genomics of Toulouse, CNRS UMR5288 Université de Toulouse, Université Paul Sabatier Toulouse France; ^4^ Museum of New Zealand Te Papa Tongarewa Wellington New Zealand

**Keywords:** ancient DNA, Haliotidae, *Haliotis*, invertebrate, Mollusca, phylogeography

## Abstract

Natural history collections worldwide contain a plethora of mollusc shells. Recent studies have detailed the sequencing of DNA extracted from shells up to thousands of years old and from various taphonomic and preservational contexts. However, previous approaches have largely addressed methodological rather than evolutionary research questions. Here, we report the generation of DNA sequence data from mollusc shells using such techniques, applied to *Haliotis virginea* Gmelin, 1791, a New Zealand abalone, in which morphological variation has led to the recognition of several forms and subspecies. We successfully recovered near‐complete mitogenomes from 22 specimens including 12 dry‐preserved shells up to 60 years old. We used a combination of palaeogenetic techniques that have not previously been applied to shell, including DNA extraction optimized for ultra‐short fragments and hybridization‐capture of single‐stranded DNA libraries. Phylogenetic analyses revealed three major, well‐supported clades comprising samples from: (1) The Three Kings Islands; (2) the Auckland, Chatham and Antipodes Islands; and (3) mainland New Zealand and Campbell Island. This phylogeographic structure does not correspond to the currently recognized forms. Critically, our nonreliance on freshly collected or ethanol‐preserved samples enabled inclusion of topotypes of all recognized subspecies as well as additional difficult‐to‐sample populations. Broader application of these comparatively cost‐effective and reliable methods to modern, historical, archaeological and palaeontological shell samples has the potential to revolutionize invertebrate genetic research.

## 1 INTRODUCTION

Invertebrates represent >95% of all animal taxa (Cowie et al., 2017; May, 1988). Mollusca is the second most diverse invertebrate phylum after Arthropoda, with approximately 200,000 species (Ponder et al., 2019), most of which produce durable carbonate shells. Indeed, hundreds of millions of mollusc shells can be found in natural history collections (NHCs) worldwide (Sierwald et al., 2018). Due to their high diversity, their prevalence in archaeological and fossil records, and the multitudes of available samples, molluscs can represent ideal study organisms to understand trends in the spatiotemporal distribution and structure of species, populations and ecosystems (Cowie et al., 2022; Foote et al., 2007; Womack et al., 2021).

Restriction of studies to recently collected ethanol‐preserved tissues greatly constrains the proportion of NHC holdings available to genetics researchers (Martin et al., 2021; Sierwald et al., 2018). Mollusc shell is a noncellular structure generally comprising both carbonate and chitinous parts (Ponder et al., 2019), and is thought to have relatively low endogenous DNA content (Martin et al., 2021). The ability to incorporate sequences from dry‐preserved mollusc shell, or those fixed in formalin (Hahn et al., 2021; Ruane & Austin, 2017) or isopropanol, greatly increases the potential ease of inclusion of type species, foreign species, topotypes (samples from the type locality), specimens from regions or habitats that are difficult to sample, and samples from endangered or recently extinct populations or taxa (Martin et al., 2021). The application of palaeogenetic techniques to mollusc shells has the potential to revolutionize evolutionary genetic research on this diverse group (Ferriera et al., 2020).

Several proof‐of‐concept studies have demonstrated the potential of mollusc shell as a DNA reservoir (reviewed in Martin et al., 2021). Success rates seem to vary significantly between samples and preservation histories (Der Sarkissian et al., 2017; Geist et al., 2008; Sullivan et al., 2021). The shells involved ranged from freshwater mussels that had been left in riverine conditions for short time periods (Geist et al., 2008) to shells that had been subjected to heating conditions (Ferriera et al., 2020), or from archaeological deposits in tropical regions (Sullivan et al., 2021), and shells with varied crystalline structures and mineralogical compositions (Der Sarkissian et al., 2017), or up to 100,000 years old (Der Sarkissian et al., 2020). However, all of these studies have used either direct PCR amplification followed by chain‐termination (i.e., Sanger) sequencing (e.g., Geist et al., 2008) or shotgun high‐throughput DNA sequencing (Der Sarkissian et al., 2017, 2020; Psonis et al., 2022; Sullivan et al., 2021). The former is labour intensive and has a high failure rate for especially fragmented DNA (Geist et al., 2008), while the latter can be prohibitively expensive depending on sample preservation. The costs associated with shotgun sequencing often mean sample size must be compromised, limiting the scope of hypotheses that can be tested. New laboratory methods for increasing the efficiency of high‐throughput sequencing of degraded DNA – namely hybridization capture enrichment (e.g., González‐Fortes & Paijmans, 2019; Horn, 2012) and single‐stranded DNA libraries (e.g., Gansauge et al., 2017) – have been widely adopted by researchers working on other taxa, but have not previously been applied to mollusc shell.

Of previous studies, only Hawk and Geller (2019), using PCR amplification and Sanger sequencing, used multiple shell‐derived DNA sequences to resolve a specific evolutionary research question (proof of application), rather than predominantly addressing methodological questions (proof of concept), and only Der Sarkissian et al. (2017, 2020), Sullivan et al. (2021) and Psonis et al. (2022) utilized high‐throughput DNA sequencing, which, without a hybridization‐enrichment stage, required a substantial and costly number of reads per sample (e.g., 4.6–56.6 million reads; Der Sarkissian et al., 2020) for complete mitogenome generation (sometimes with limited nuclear data also reported).

**FIGURE 1:**
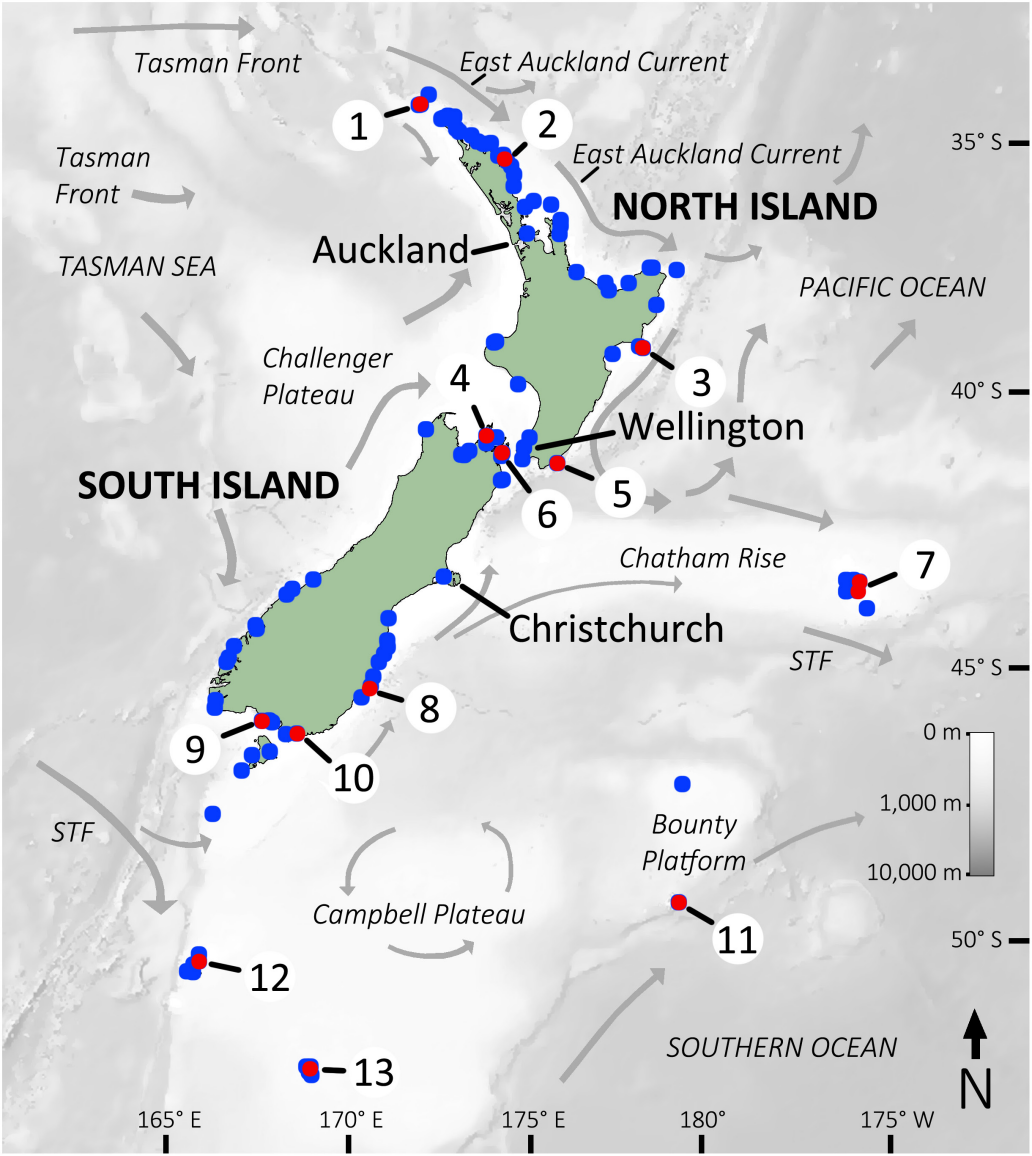
Map of New Zealand. Coloured circles denote distribution records of *Haliotis virginea* held at the Museum of New Zealand Te Papa Tongarewa and the National Institute of Water and Atmospheric Research, both in Wellington, New Zealand; red = samples sequenced in this study (Table 1). Grey arrows (size not significant) depict generalized surface current directionality loosely adapted from Chiswell et al. (2015) and Stevens et al. (2021). STF, subtropical front. (1) Three Kings Islands. (2) Bay of Islands, the type locality of *H. virginea crispata* A. Gould, 1847. (3) Mahia Peninsula. (4) Pelorus Sound, Marlborough Sounds. (5) Wairarapa coast. (6) Tory Channel, Marlborough Sounds; Tory Channel is a subsidiary of Queen Charlotte Sound, which is the type locality of *H. virginea* Gmelin, 1791. (7) Chatham Islands; Owenga, on the east of the main island, is the type locality of *H. virginea morioria* Powell, 1938. (8) Otago Harbour. (9) Kawakaputa Bay, W of Invercargill, Southland. (10) Waipapa Point, Catlins coast, Southland. (11) Antipodes Islands, the type locality for *H. virginea stewartae* M. Jones & B. Owen, 2004. (12) Auckland Islands. (13) Campbell Island, the type locality of *H. virginea* huttoni Filhol, 1880.

*Haliotis virginea* Gmelin, 1791 is a medium‐sized (to 75 mm, Geiger & Owen, 2012) species of abalone (family: Haliotidae) that occurs on rocky coasts around the New Zealand region (Figure 1), and which grazes on algae from the intertidal zone to about 50 m depth. Spatially structured phenotypic variation has led to the contentious recognition of five subspecies (Geiger & Owen, 2012; Jones & Owen, 2011; Owen & Kershaw, 2014) in addition to other regional forms. However, many morphological characters vary clinally, complicating the classification of some populations as subspecies. In this study, we use near‐complete mitogenomes from a combination of ethanol‐preserved tissues and historically collected dry‐preserved shells to test whether the molecular phylogeny of *H. virginea* recapitulates the distinctiveness and distribution of previously identified subspecies and forms. *H. virginea* is an ideal study organism for a proof‐of‐application of palaeogenetic methods in molluscan phylogeography because it has a broad distribution throughout New Zealand (Figure 1), shell samples from throughout this distribution are available in NHCs, and evidence suggests that haliotids are well‐suited for shell‐derived DNA sequencing (Der Sarkissian et al., 2017; Hawk & Geller, 2019).

## 2 MATERIALS AND METHODS

### 2.1 Ethanol‐preserved tissues

Samples (Table 1) were collected by turning boulders at low tide, or by SCUBA from shallow subtidal depths, and were preserved in 98% ethanol. Vouchers were lodged at the Museum of New Zealand Te Papa Tongarewa (NMNZ, 6‐digit registration numbers prefixed ‘M.’) in Wellington, New Zealand, from where additional samples were also sourced.

**TABLE 1:** Samples used in phylogenetic analyses

Voucher	Locality	Collected	Taxon/form	GenBank ID
M.059511 #	Great Island, Three Kings Islands	1976	Three Kings	ON990040
M.100393 #	Great Island, Three Kings Islands	1988	Three Kings	ON990041
M.333468 (a)	Great Island, Three Kings Islands	2020	Three Kings	ON990038
M.333468 (b)	Great Island, Three Kings Islands	2020	Three Kings	ON990039
M.004673 #	Russell, Bay of Islands, Northland	–	*crispata*	ON990057
M.333473 #	Auroa Pt, Mahia Peninsula, Hawkes Bay	2017	*crispata*	ON990049
M.318759	SE of Pahaoa, Wairarapa coast	2015	Nominate *s. l*.	ON990055
M.134010 #	Tory Channel, Marlborough Sounds	1996	Nominate *s. s*.	ON990050
M.129796	Pelorus Sound, Marlborough Sounds	2015	Nominate *s. s*.	ON990051
M.333472 (a)	Dowling Bay, Otago Harbour, Otago	2020	Nominate *s. l*.	ON990036
M.333472 (b)	Dowling Bay, Otago Harbour, Otago	2020	Nominate *s. l*.	ON990037
M.317709 (a)	Waipapa Pt, Catlins coast, Southland	2014	Nominate *s. l*.	ON990052
M.317709 (b)	Waipapa Pt, Catlins coast, Southland	2014	Nominate *s. l*.	ON990053
M.317709 (c)	Waipapa Pt, Catlins coast, Southland	2014	Nominate *s. l*.	ON990054
M.285403	W of Invercargill, Southland	2009	Nominate *s. l*.	ON990056
M.333471 #	Ewing Is, Port Ross, Auckland Islands	2018	*huttoni*	ON990048
M.117477 #	South Pt, Campbell Island	1991	*huttoni*	ON990059
M.117609 #	Perseverance Harbour, Campbell Island	1985	*huttoni*	ON990058
M.333469 #	WNW of Owenga, Chatham Islands	2013	*morioria*	ON990047
M.333470 #	S of Point Munning, Chatham Islands	2013	*morioria*	ON990042
NIWA 107994 (a) #	Antipodes Islands	1962	*stewartae*	ON990043
NIWA 107994 (b) #	Antipodes Islands	1962	*stewartae*	ON990044
N/A	“Christchurch coast”, New Zealand	–	*Haliotis iris* (outgroup)	KU310895. Origin: Guo, Jiang, et al. (2019)
M.333474	Perth, Western Australia, Australia	2019	*Haliotis scalaris* (outgroup)	ON990045
M.333475	Perth, Western Australia, Australia	2019	*Haliotis semiplicata* (outgroup)	ON990046
Museo Nacional de Ciencias Naturale, Madrid, Spain: ADN 85530	Cabo de Palos, Murcia, Spain	–	Fissurellidae: *Diodora graeca* (outgroup)	KT207825. Origin: Uribe et al. (2017)

*Note*: “#” indicates shell‐derived sequences. GenBank IDs prefixed “ON”, were generated by this study. Letters in parentheses denote distinct individuals from the same multispecimen lot where applicable. Voucher numbers prefixed “M.” are held by the National Museum of New Zealand, Wellington, New Zealand. NIWA refers to samples held by the National Institute of Water and Atmospheric Research, Wellington, New Zealand. Taxon/Form refers to *Haliotis virginea* unless specified as “outgroup”. “Collected” refers to year of collection (if known).

DNA was extracted using a MagAttract high molecular weight DNA Kit (Qiagen), following the manufacturer's instructions. Custom long‐range PCR primers (Table 2) from conserved regions were designed in geneious prime version 2021.1 (Biomatters); using haliotid mitogenomes downloaded from GenBank (accession numbers: FJ599667, KF724723, KU310895, KX260956, NC005940) and aligned in geneious prime using the Geneious Alignment function with default settings. The four resulting mostly‐overlapping amplicons essentially covered the entire haliotid mitogenome except for the Control Region. Long‐range PCRs contained: 1× PrimeSTAR GXL Buffer (Takara), 0.2 μM dNTPs, 0.2 mM each of forwards and reverse primer, 0.025 U/μl PrimeSTAR GXL DNA Polymerase (Takara) and 50–100 ng of DNA template. Thermocycling conditions comprised a 10 s initial denaturation at 98°C followed by 30 cycles of 98°C for 10 s, 55°C (primer pairs 1, 2 and 4) or 57°C (primer pair 3) for 15 s, then 68°C for 1 min/kb. A final extension stage followed of 1 min/kb at 68°C. PCR products were visualized on an agarose gel to confirm amplification success.

**TABLE 2:** Details of the custom long‐range primers used in the amplification of haliotid mitogenomes

Primer name	Primer pair	Approx. frag. length (kb)	Gene location	Primer sequence (5′–3′)
Hal_F_1	1	8.3	tRNA‐Asn	CCT GAC TGT TAA GGA GAC TG
Hal_R_1			tRNA‐Phe	GAA GTG TTC GAA TCA CTT AGT AGC
Hal_F_2	2	5.4	tRNA‐Thr	TCG GTC TTG TAA GCC GAA GG
Hal_R_2			12S	ACT GCG GTT AGA CAA ACA GG
Hal_F_3	3	1.0	12S	ACT CCG AAT AAC GGG ATA CC
Hal_R_3			tRNA‐Glu	GAG CAC GTT AGG TTT TCG TC
Hal_F_4	4	1.6	CO3	GAC CCG AAG ACC TTT TCA TC
Hal_R_4			tRNA‐Ser	AAA CAA AGT TAG CAG CCC TG

Amplicons were fragmented to 150–400 bp with a Picoruptor (Diagenode) sonicator at 4°C using 13 cycles of: 30 s sonication then 30 s rest. Fragmented amplicons were purified using AMPure XP carboxyl‐coated magnetic beads (Agencourt) at 1.8:1 (bead‐mix:template ratio) to remove unincorporated PCR reagents and DNA fragments <~50 bp in length. Purified, sheared amplicons were quantified using a Qubit version 3.0 fluorometer (Qiagen) dsDNA Broad Range Assay kit (Qiagen).

Double‐stranded Illumina sequencing libraries were prepared following the methods reported in Meyer and Kircher (2010). The optimal number of indexing cycles (#_o_ – ranges: 9–12 and 12–22 for ethanol‐preserved tissues and dry‐preserved shells, respectively) was established with quantitative PCR (qPCR) to reduce clonality and limit heteroduplex formation (Gansauge et al., 2020). Each 10 μl qPCR contained: 1× Maxima SYBR Green qPCR Master Mix (Thermo Scientific), 0.2 μl each of IS7 and IS8 primer (Meyer & Kircher, 2010), and 0.1 μl of library. A QuantStudio 5 Real‐Time PCR System was used with the thermocycling conditions: 95°C for 10 min; and 40 cycles of 95°C for 30 s, 60°C for 30 s and 72°C for 30 s. P5/P7 adapters with unique 7‐mer barcode combinations (Scarsbrook et al., 2022) were added to libraries through indexing PCRs (four reactions per library) containing: 1× High Fidelity PCR Buffer (Invitrogen), 2 mM MgSO_4_, 0.25 mM dNTPs, 1.25 U Platinum Taq DNA Polymerase High Fidelity (Invitrogen), 0.5 μM of each indexing primer (Meyer & Kircher, 2010) and 5 μl of library. Thermocycling conditions were: an initial denaturation phase of 94°C for 12 min; #_o_ cycles of 94°C for 30 s, 60°C for 30 s, then 72°C for 45 s; then a final extension of 72°C for 10 min. Replicate indexed libraries were pooled, purified and quantified as above. Library fragment size distributions were determined using a Fragment Analyser (Agilent) and prosize software version 2.0 (Advanced Analytical Technologies). Libraries were diluted to 10 nM, pooled in equimolar concentrations, and sequenced using an Illumina MiSeq (Otago Genomics) with 2 × 150 bp (paired‐end) sequencing chemistry.

Adapter sequences were removed and paired‐end reads merged using adapterremoval version 2.3.1 (Schubert et al., 2016). Low‐quality bases were trimmed (‐‐minquality: 30; ‐‐trimns) and collapsed reads <25 bp were discarded (‐minlength: 25). Read quality was visualized using fastqc version 0.11.9 (Andrews, 2010). Mitogenomes were assembled in geneious prime through mapping collapsed reads against a published *Haliotis iris* Gmelin, 1791 mitogenome (KU310895, Table 1) using the Geneious Read Mapper with medium‐sensitivity parameters.

### 2.2 Dry‐preserved shell

Dry‐preserved shell samples (*n* = 12; Table 1) used ranged in age from 4 to 60 years (mean: 32.1) since collection (the animals potentially having died years prior). Some were newly sourced from beaches; others from NMNZ and the National Institute of Water and Atmospheric Research (NIWA, Wellington, New Zealand). The preservation histories of M.059511 and M.100393 from the Three Kings Islands are uncertain; these may have been in isopropanol. The oldest samples, collected in 1962, were the two Antipodes Islands shells (NIWA 107994), which are believed to have been fixed in isopropanol then stored dry. All other samples have not been in fixative and were stored in cool, stable conditions since collection. Surface contamination was removed through immersion in 5% bleach for 5 min with encrustations (biofouling) visible to the naked eye removed using a dental pick prior to lab entry. Note that bleach pretreatment may damage/alter voucher specimens.

Shell samples were processed in the Otago Palaeogenetics Laboratory at the University of Otago. This facility is dedicated to the handling of historical and ancient samples believed to have low DNA content. Strict anticontamination protocols were followed (Knapp et al., 2012). A Dremel cutting tool was used to subsample approximately 50 mg of shell from a clean‐looking part of the growing edge of the shell aperture, which was the most recently deposited and, generally, the least encrusted part of the shell. Subsamples were ground to a fine powder using a mortar and pestle. Between 10 and 50 mg of powdered shell was added to 1 ml of digestion solution comprising 0.45 M EDTA pH 8.0, 0.25 mg/ml Proteinase K and 0.05% Tween20, and the solution then incubated for 12–48 h at 37°C under constant rotation. Remaining shell material was pelleted by centrifugation with supernatant subjected to a spin‐column DNA extraction method following Dabney et al. (2013).

Success of initial DNA extractions was confirmed by PCR amplification of a 73 bp (excluding primers) section of the mitochondrial gene CO1 using custom primers. Each 10 μl reaction contained 1× PCR buffer II (ThermoFisher), 2 mM MgCl_2_, 0.16 mg/μl of BSA (ThermoFisher), 0.4 mM each of forward (5′‐ATY TGA TCC GGC YTA GTY GGA AC‐3′) and reverse (5′‐GCR TGR GCT GTT ACA ATT AC‐3′) primer, 0.2 mM dNTPs, 2 U/μl of AmpliTaq Gold (ThermoFisher) and 1 μl of unquantified DNA extract template (or water for PCR negatives). Thermocycling conditions were a 120 s initial denaturation at 94°C followed by 45 cycles of: 94°C for 60 s, 54°C for 60 s then 72°C for 90 s; then a 120 s final extension at 72°C. PCR products were visualized on agarose gel to confirm amplification. Samples were Sanger sequenced by Genetic Analysis Services (University of Otago, Dunedin, New Zealand) and the GenBank blast function (default settings) used to confirm amplified DNA was on target (i.e., haliotid CO1).

Preliminary shotgun sequencing data (not reported; available at: Walton et al., 2022, https://doi.org/10.5061/dryad.3ffbg79mq) generated from double‐stranded Illumina sequencing libraries created following the single‐tube protocol of Carøe et al. (2018) yielded a very low proportion (<0.1%, Table S4) of target (haliotid mitogenomic) reads. Accordingly, we employed a single‐stranded Illumina sequencing library approach (Gansauge et al., 2017; described in full in Scarsbrook et al., 2022, supporting information methods “C”) to improve recovery of highly fragmented DNA. Quantitative and indexing PCRs were carried out as above. Extraction negatives did not amplify on test PCRs and returned delayed amplification curves equivalent to those of library preparation negatives in qPCRs.

Bait generation and hybridization capture methods (described in full in: Scarsbrook et al., 2022) were adapted from Maricic et al. (2010), Horn (2012) and González‐Fortes and Paijmans (2019) to selectively enrich haliotid mitogenomic DNA and to reduce the number of off‐target (i.e., exogenous) sequences recovered, such as from shell‐boring organisms. Biotinylated DNA baits were generated from long‐range PCR product from an ethanol‐preserved mainland specimen of *H. virginea* (M.333472, Table 1). Hybridization reactions contained: 1× hybridization buffer and blocking agent (both: Agilent), 2 μM each of BO1.P5.F and BO1.P7.F custom blocking oligos (Scarsbrook et al., 2022) and a 5:1 ratio of library to bait mix, and were incubated for 48 h at 55°C.

Each 25 μl post‐capture reamplification PCR contained: 1× AmpliTaq Gold Buffer (ThermoFisher), 2.5 mM MgCl_2_, 0.25 mM dNTPs, 1.25 U AmpliTaq Gold DNA Polymerase (ThermoFisher), 0.5 μM each of IS5 and IS6 primer (Meyer & Kircher, 2010), and 5 μl library. Thermocycling conditions were: 94°C for 12 min; #_o_ cycles of 94°C for 30 s, 60°C for 30 s and 72°C for 45 s; and 72°C for 10 min. Reamplified libraries were purified and quantified for equimolar pooling as above. Mean library fragment sizes were measured with a QIAxcel Advanced System using a 25–10,000 bp alignment marker. Data calibration and visualization were performed with qiaxcel screengel Software version 1.6.0 (Qiagen). Each library was diluted to 10 nM. Libraries were pooled and run on an Illumina NextSeq (Garvin Institute of Medical Research, Darlinghurst, Australia) using 2 × 75 bp (paired‐end) sequencing chemistry and custom sequencing primers (“CL72_custom”: 5′‐ACA CTC TTT CCC TAC ACG ACG CTC TTC C‐3′, Gansauge & Meyer, 2013; and “G'stein_custom”: 5′‐GGA AGA GCG TCG TGT AGG GAA AGA GTG T‐3′, Paijmans et al., 2017).

Mitochondrial sequence alignments were generated using the BAM pipeline implemented in paleomix version 1.2.14 (Schubert et al., 2014). Specifically, adapter sequences and low‐quality bases (‐‐minquality: 30; ‐‐trimns) were trimmed in adapterremoval version 2.3.1 (Schubert et al., 2016), with efficacy assessed using fastqc version 0.11.9 (www.bioinformatics.babr‐aham.ac.uk/projects/fastqc/). Resulting collapsed reads were mapped against a published *H. iris* mitogenome (KU310895, Table 1) using BWA version 0.7.17 (‐n: 0.01; ‐o: 2) (Li & Durbin, 2009). samtools version 0.1.19 (‐q: 25) (Li et al., 2009) was used to filter reads with a mapping quality Phred score > 25, with duplicates discarded using the MarkDuplicates.jar tool in picard version 2.1.0 (https://broadinstitute.github.io/picard/). DNA damage patterns (Figure S1) characteristic of historic/ancient samples were assessed using mapdamage version 2.0.8 (Ginolhac et al., 2011; Jónsson et al., 2013) and linear regression (Figure S2) performed on the outputs in the R statistical environment version 3.6.1 (R Development Core Team, 2019) to assess temporal trends. Misalignments from reads overlapping indels were improved using the IndelRealigner tool in gatk version 4.1.4.1 (McKenna et al., 2010). Majority consensus sequences (75%) were generated for each BAM alignment in geneious prime, with bases only called at sites covered by ≥3 reads (with IUPAC ambiguities otherwise called).

### 2.3 Alignment and phylogenetics

Outgroup reference mitogenomes were downloaded from GenBank (Table 1). Initial alignments and gene directionality were established in geneious prime using the Geneious Alignment function under default parameters with the following modifications: a gap‐opening penalty of 30 and a 65% similarity Cost Matrix. Near‐complete mitogenome alignments were approximately 16 kb in length and were uploaded to GenBank (accession nos: ON990036–ON990059). For downstream phylogenetic analysis, we extracted and independently realigned protein‐coding gene sequences using the Translational Alignment function (geneious prime) with the blosum62 cost matrix and default parameters (including free end gaps) to ensure homologous coding‐frame comparisons. ATP8 and ND2 were excluded as the outgroup could not be convincingly aligned at those positions. This final alignment can be found at Dryad (Walton et al., 2022; https://doi.org/10.5061/dryad.3ffbg79mq) and comprised 10,059 bp (9993 bp for *H. virginea* without gaps).

We used partitionfinder version 1.0.1 (Lanfear et al., 2012) to determine the optimal partitioning scheme and substitution models for downstream phylogenetic analyses. We defined six putative partitions, corresponding to the first, second and third codon positions for the protein coding genes encoded on the heavy strand (H‐strand: ND1, ND6, CYTB, ND4L, ND4, ND5) and light strand (L‐strand: ATP6, COX2, COX1, ND3, COX3). The best partitioning scheme according to the Bayesian Information Criterion (as implemented in partitionfinder) supported independent models for each partition: H‐strand first codon position (GTR+I+G), H‐strand second codon position (GTR+G), H‐strand third codon position (GTR+G), L‐strand first codon position (SYM+I), L‐strand second codon position (GTR+G), and L‐strand third codon position (SYM+G).

Maximum‐likelihood and Bayesian phylogenies were generated using iq‐tree version 1.6.11 (Nguyen et al., 2015) and mrbayes version 3.2.7a (Ronquist et al., 2012), respectively. In iq‐tree, data was partitioned such that each partition had its own unspecified evolutionary rate, but all partitions contributed to the branch lengths of a single best tree (‐spp; Chernomor et al., 2016), with topological support assessed using 1000 ultrafast‐bootstrap replicates (resampling within partitions; Hoang et al., 2017). Similarly, substitution models and rates were estimated separately for each partition in mrbayes, but all partitions informed the topology and branch lengths of a shared tree. Four separate Markov chains (one cold and three incrementally heated) were run with default parameters using the CIPRES Science Gateway version 3.3 (www.phylo.org/; UC San Diego, USA). Each chain ran for 10^6^ generations, sampling every 10^3^. Topological convergence was assessed by monitoring the average standard deviation of clade frequencies (<0.02), while convergence of individual parameter values was ensured by ensuring effective sample sizes (ESS) of >200 as calculated in tracer version 1.7.1 (www.tree.bio.ed.ac.uk/software/tracer/). Sampled trees were summarized as a majority‐rule consensus tree after discarding the first 10% of trees as burnin. figtree version 1.4.4 was used to re‐root and generate phylogenetic tree image files. popart (Leigh & Bryant, 2015) was used to generate a minimum‐spanning (Bandelt et al., 1999) haplotype network map. Figures were rendered in photoshop CS5 (Adobe). Raw percentage sequence divergence values between individuals and clades were calculated in geneious prime using default parameters.

## 3 RESULTS

In total, 22 near‐complete (lacking only the Control Region and the adjacent ends of the gene COX3 and tRNA‐Glu, Table S1), high coverage (means of 95–488 and 55–829 reads/site derived from shell and tissue samples, respectively; Table S1) mitogenomes were generated for *H. virginea*. These mitogenomes represented seven morphological forms and included all five currently recognized subspecies (Table 1). Mitochondrial gene arrangement and transcriptional direction for *H. virginea* were the same as previously reported in *H. iris* (Guo, Jiang, et al., 2019). We do not observe regions of very low coverage in our mapping results that might indicate gene rearrangements from the reference mitogenome.

All 12 DNA extractions from dry‐preserved shell were sequenced successfully. Double‐stranded DNA libraries for nine of those 12 samples were also screened on a preliminary MiSeq run (not reported; available at: Walton et al., 2022, https://doi.org/10.5061/dryad.3ffbg79mq), which yielded a mean haliotid endogenous mitogenomic content of 0.005% (Table S4). Resulting from both our transition to a single‐stranded approach and the addition of a hybridisation‐capture enrichment step, these nine samples showed a >350‐fold mean increase of haliotid endogenous mitogenomic content to 0.919% (mean) of mapped reads (Table S4). Characteristic damage patterns authenticated the recovered mapped haliotid mitochondrial DNA as degraded, and therefore unlikely to reflect contamination from more recent samples. Linear regression revealed a moderate (*R* ^2^ = .342) and significant negative relationship between C‐to‐T substitution frequency (at 5′ read ends) and sample collection date (*F*_1,10_ = 6.705, *p* = .027; Figure S2, Table S2).

**FIGURE 2:**
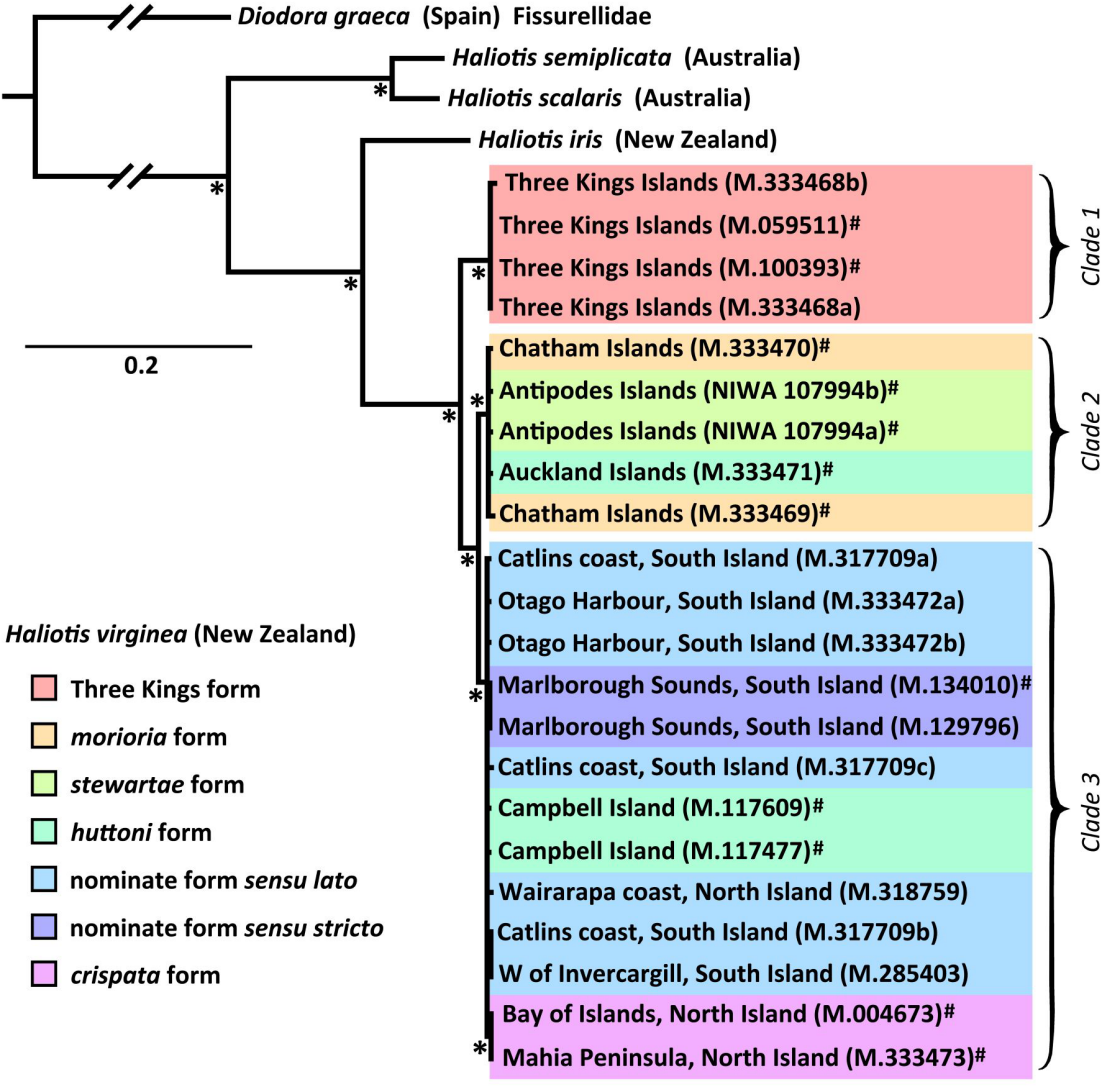
Maximum likelihood (ML) phylogenetic tree showing relationships in *Haliotis virginea* generated in iq‐tree using a 10,059 bp (9,993 bp without gaps for *Haliotis* spp.) concatenated alignment of all mitochondrial protein‐coding genes except ATP8 and ND2. A Bayesian tree generated from the same alignment in mrbayes had homologous topology (Figure S3). “*” denotes ML consensus node bootstrap support of ≥99% and Bayesian posterior probabilities of 1. “#” denotes sequences sourced from dry‐preserved shell. Scale represents substitutions per site.

**FIGURE 3:**
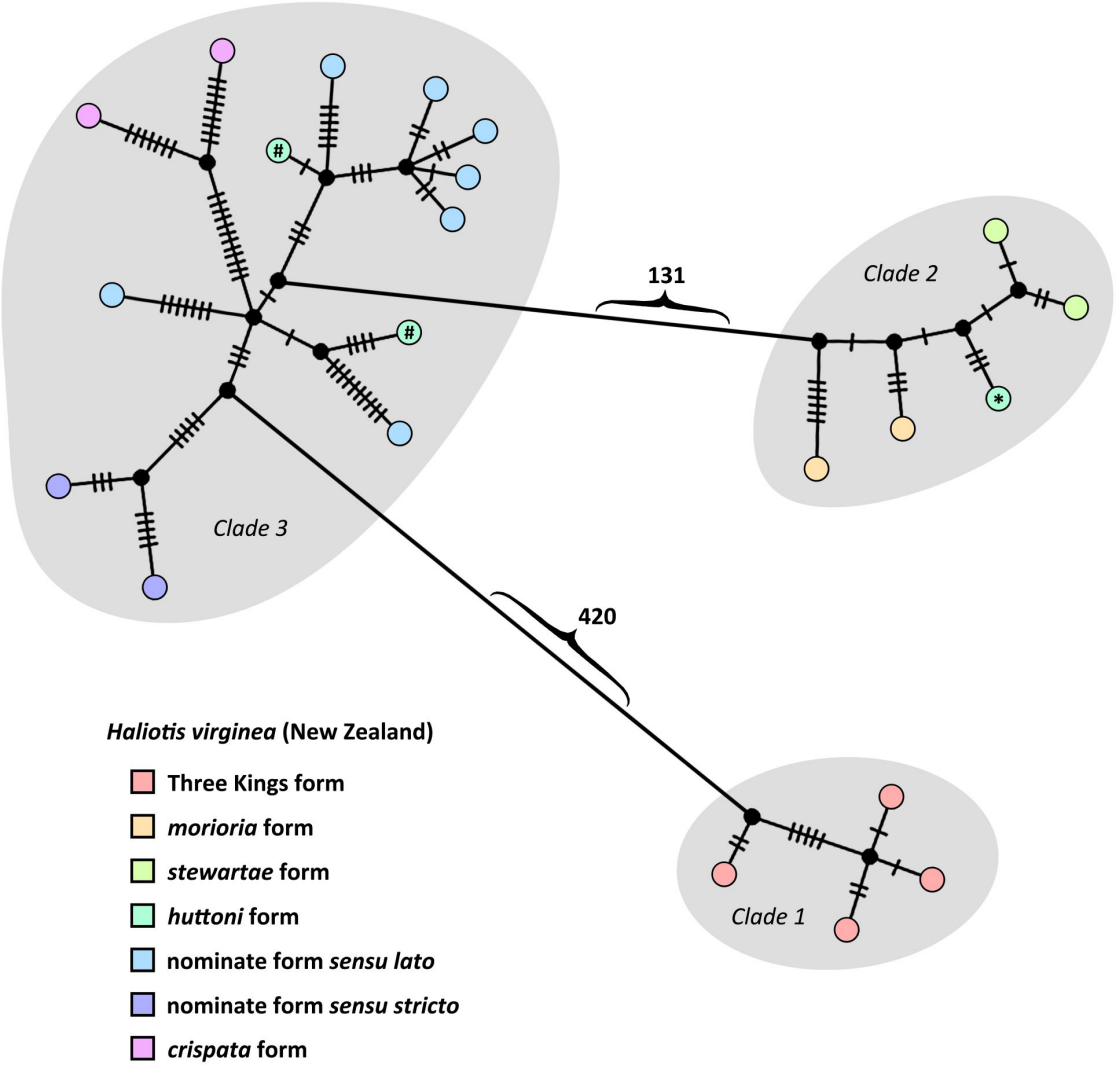
Median joining haplotype network map for *Haliotis virginea* generated in popart using a 9,993 bp concatenated alignment of all mitochondrial protein‐coding genes except ATP8 and ND2. Hatch‐marks indicate number of mutational steps between haplotypes; numbers indicate number of steps between clades identified in our phylogenetic analyses (grey shading). Black circles indicate hypothetical unsampled haplotypes; coloured circles indicate one sample. “#” and “*” indicate *H. virginea* huttoni samples from Campbell and Auckland Islands, respectively. An annotated version with sequence IDs is available in Figure S4.

From the approximately 10 kb data set analysed, three major, well‐supported (all with ≥99% bootstrap support and Bayesian posterior probabilities of 1) *H. virginea* clades were recovered: (Clade 1) Three Kings Islands; (Clade 2) Auckland, Chatham and Antipodes Islands; and (Clade 3) mainland New Zealand and Campbell Island (Figures 2 and 3; Figure S3). Clades 2 and 3 were sister (minimum sequence divergence of 1.95%) to the exclusion of Clade 1 (Figure 2; Figure S3). Clade 1 had a sequence divergence of 4.3%–4.5% from Clades 2 and 3. A CO1 alignment (532 bp aligned with the start of the *Haliotis rugosa pustulata* Reeve, 1846 sequence AY923918) recovered these same three clades, with sequence divergence values observed between Clade 1 and Clades 2 and 3 of 4.7% and 3.9% respectively: figures which are greater than those between several widely recognized *Haliotis* species (Table S3).

Clade 1 (Three Kings Islands) represented the only monophyletic morphological form of *H. virginea* that was highly divergent from other recognized forms. Within Clade 2, which included topotypes of *H. virginea morioria* Powell, 1938 and *H. virginea stewartae* M. Jones & B. Owen, 2004, as well as the Auckland Islands sample of *H. virginea huttoni* Filhol, 1880, there was relatively little sequence divergence or structure corresponding to these three subspecies (Figures 2 and 3). Within Clade 3, which contained near‐topotypic samples of nominate *H. virginea* as well as topotypes of *H. virginea crispata* A. Gould, 1847 and *H. virginea huttoni*, there was similarly little divergence or deep structure corresponding to these three forms. However, the two near‐topotypic samples of nominate *H. virginea* as well as the two *H. virginea crispata* samples were respectively paired together as well supported (Bayesian posterior probability values of 1, bootstrap support values of 99%) monophyletic subclades (Figures 2 and 3).

## 4 DISCUSSION

Our study demonstrates that generation of mitogenomes from dry‐preserved historical mollusc shells is not merely possible (Der Sarkissian et al., 2017, 2020), but can be consistently achieved, cost‐effective, and practicable to address research questions. All samples that were attempted yielded high‐coverage near‐complete mitogenomes, allowing the testing of phylogeographic hypotheses across a broad geographic range utilizing specimens of differing ages and preservational histories. Mitogenomes were generated from small amounts (10–50 mg) of powdered shell compared to methods reported by Der Sarkissian et al. (2017, 2020) and Sullivan et al. (2021), which used as much as 1 g of powdered shell per sample: most mollusc species do not exceed 1 g at maturity. DNA degradation correlated with sample age despite a relatively narrow age range in the historical samples used (Figures S1 and S2), highlighting the need for palaeogenetic approaches, even for recently collected mollusc shells. However, for even the oldest (60 years since collection) samples, this approach yielded high‐quality data. Accordingly, it seems likely that the combination of approaches reported here can be applied to much older samples, such as those from archaeological or palaeontological contexts.

While the effects of preservation history and taxonomic biases on success rates of shell‐derived DNA sequencing remain to be well understood (Der Sarkissian et al., 2017; Martin et al., 2021), it seems probable that similar approaches to those reported here can be applied to a broad variety of historical, archaeological and subfossil mollusc material in NHCs. If so, the proportion of NHC holdings that can be expected to practicably yield DNA sequence data might increase by orders of magnitude, thereby facilitating numerous lines of research previously limited by availability of suitable samples. For example, at the commencement of this project, ethanol‐preserved samples were available for only one of the five currently recognized subspecies, and two of the seven forms we note here (Figures 2 and 3, Table 1).

Critically, our approaches remove the need for costly and/or unreliable re‐sampling from difficult to access locations, such as Antarctic, alpine or deep‐sea faunas (Orr et al., 2020), or of endangered (Geist et al., 2008; Hawk & Geller, 2019), recently extinct (Geist et al., 2008; Sullivan et al., 2021) or difficult‐to‐find (Psonis et al., 2022) populations and taxa. Use of shell‐derived DNA sequences would, furthermore, enable easier inclusion of topotypic samples and type taxa in data sets, in addition to reducing the need to source fresh samples from many institutions and countries (and the associated cost and time of doing so).

Mollusc shell‐derived palaeogenetic approaches might form entire data sets or supplement otherwise “conventionally” derived data sets, as reported here. Additionally, these approaches, or parts thereof, might be applied to other carbonate‐structure‐producing invertebrate groups, such as bryozoans (Orr et al., 2020), crustaceans, echinoderms, or corals (Gomez Cabrera et al., 2019). However, bait generation may be more costly and time consuming for phyla with fewer available reference sequences (Orr et al., 2020), as the additional step of Sanger‐sequencing amplicons generated using “universal primers” may be necessary to develop effective taxon‐specific long‐range PCR primers. These enrichment approaches might also be applied to generation of nuclear DNA data (Meyer et al., 2016), especially given the low endogenous mitochondrial DNA content in even comparatively modern *Haliotis* specimens. However, relatively few nuclear reference genomes, which would improve both the design of capture‐based approaches as well as mapping of shotgun data, are presently available for invertebrates. Due to signatures of DNA degradation (i.e., base pair modification, fragmentation) and low levels of endogenous DNA observed in even relatively “modern” samples (Figure S1; see also Der Sarkissian et al., 2017, 2020; Ferriera et al., 2020), use of a specialized palaeogenetics facility is strongly recommended to avoid exogenous contamination (Cooper & Poinar, 2000; Knapp et al., 2012).

The ability to source foreign samples more easily (i.e., from local NHCs), however, further removes sample oversight and data‐sovereignty matters from governments and indigenous communities in their country of origin. Thus, application of these methods places greater onus on researchers and NHC staff to ensure adherence to ethical and culturally appropriate research practices (a hotly debated area; see Carroll et al., 2020; Liggins et al., 2022). Our study also emphasizes the significant unrealized value NHCs can have as archives of all manner of data to address yet‐to‐be asked or thought of research questions (Sheperd & Perrie, 2012).

### 4.1 Phylogeography of *Haliotis virginea*

Topotypic samples were selected to closely resemble the actual primary types of each subspecies to best ensure that our sequences are representative of those potential taxa. Paraphyly in *H. virginea huttoni*, comparatively low genetic distances between many of the currently recognized subspecies of *H. virginea* (Figures 2 and 3), and intergrading and/or overlapping morphological variation between several subspecies (see below), indicate that their continued recognition may not be justified. Conversely, the Three Kings Islands population may warrant naming as a distinct taxon, given the considerable genetic distance between it and all other sampled populations (Table S3). There is extraordinarily high species‐level endemism in the coastal marine fauna at the Three Kings (Marshall & Marrow, 2021), and Three Kings specimens of *H. virginea* consistently but subtly differ morphologically in several aspects of shape and sculpture from those from northern parts of mainland New Zealand. Mainland populations intergrade clinally, which had led previous writers to suggest the taxonomic distinction between nominate *H. virginea* and *H. virginea crispata* be discontinued (Jones & Owen, 2011; Walton, 2014).

Weak‐to‐moderate phylogeographic structure has been reported in *H. iris*, the presumed sister‐species of *H. virginea* (Degnan et al., 2006; Figure 2), with phylogeographic breaks occurring: (1) in the vicinity of Cook Strait, separating “northern” and “southern” mainland populations; and (2) between mainland populations and those on the Chatham Islands (Smith & McVeagh, 2006; Will et al., 2011). These reported phylogeographic disjunctions in *H. iris* conform roughly to reported morphological differences in the shells of *H. virginea* (namely, nominate *H. virginea* from the South Island, *H. virginea crispata* from the North Island and *H. virginea morioria* from the Chatham Islands). All abalone are broadcast spawners (Wilson & Schiel, 1995). Larval characteristics for *H. virginea* are not well understood, although they are likely to be similar to those of related taxa. The larvae of *H. iris* disperse passively in the water‐column (McShane, 1992), generally for 5–9 days (Tong et al., 1992). However, they can delay metamorphosis for at least 3 weeks (Roberts & Lapworth, 2001). Surface currents (Figure 1) might therefore be expected to result in directional connectivity between proximate populations.

Discrete morphological differences in populations of *H. virginea* from the southern islands (south of 43°S) of New Zealand may reflect the absence of suitable habitat between islands as well as ecophenotypic effects of local environmental conditions and algal (food) communities. A diverse range of distribution patterns of coastal marine mollusc species occur in the southern islands of New Zealand, making biogeographic generalizations impractical. Many taxa formerly considered to be restricted to the New Zealand subantarctic islands proved to be synonymous with populations from the southern South Island following genetic studies (Nikula et al., 2013; Reisser et al., 2011; Salloum et al., 2020).

The coastal phylogeography and biogeography of the southern islands of New Zealand south of 43°S is driven by several factors. The generally eastern flow through the region of the Subtropical Front (Figure 1; Chiswell et al., 2015; Stevens et al., 2021), which represents a confluence of relatively warm and cool bodies of water, serves as a biogeographic barrier to many marine species (Compton et al., 2013; Hollis & Neil, 2005). Conversely, rafting of benthic organisms associated with the large, abundant, buoyant alga *Durvillaea antarctica* (Chamisso) Hariot, 1892 has resulted in elevated levels of connectivity over long distances between the southern islands of New Zealand (and beyond) within several marine species (Fraser et al., 2011; Nikula et al., 2011a, 2011b). Garden and Smith (2011) reported that *Durvillaea* rafts can transport rocks of tens to potentially hundreds of kilograms. Although *H. virginea* is not normally directly associated with kelp holdfasts, the species co‐occur in high abundance, implying that infrequent rafting events are inevitable.

Too few samples were sequenced to understand how the Auckland Islands sample of *H. virginea huttoni* came to fall within the Clade 2, rather than with the morphologically very similar Campbell Island samples that placed in Clade 3, or if this relationship is reflective of the Auckland Islands population generally. Our result may reflect past rafting and/or introgression events, or incomplete lineage sorting, which can result in discordant phylogenetic signals among different markers (e.g., Funk & Omland, 2003). Indeed, the cause and timing of the moderate divergence between Clades 2 and 3 is also unclear in the absence of reliable phylogenetic calibration points or evolutionary rates within this family.

Despite significant isolation, the coastal marine mollusc fauna of the Chatham Islands has surprisingly low levels of regional species‐level endemism (Walton et al., 2018). *H. virginea morioria* from the Chatham Islands most closely resemble mainland forms. However, in some characters and specimens (not those sequenced here, which resembled the holotype), Chatham Islands shells also share a resemblance to *H. virginea stewartae* from the Bounty Plateau, which is the most morphologically distinctive of all *H. virginea* subspecies.

Our study did not include nuclear markers. There are presently no reference nuclear genomes available for haliotids or closely related taxa. Mitogenomes are usually uniparentally inherited, have lower effective population sizes relative to nuclear markers, and are effectively inherited as a single locus that may not be under neutral selection (Ballard & Whitlock, 2004). Mitochondrial data can sometimes be misleading due to a variety of factors, such as introgression, substitution saturation, infrequent interpopulation admixture, and incomplete lineage sorting (Funk & Omland, 2003; Phillips & Zakaria, 2021; Wort et al., 2017). These and other drawbacks of mitogenomes over nuclear markers, have led some authors to question the value of mitogenomes in phylogeographic analyses (e.g., Ballard & Whitlock, 2004). However, mitogenomes are relatively cost‐efficient and generally provide a high degree of resolution to address phylogeographic questions (Phillips & Zakaria, 2021). Haliotids have an unusual (for molluscs) propensity to hybridize readily with other, sometimes quite distantly related species, which has the potential to generate misleading signals in mitochondrial data (Guo, Ding, et al., 2019; Hirase et al., 2021). There was no indication of hybridization with out‐group taxa within our data set, which could be characterized by polyphyly in the in‐group (*H. virginea*) due to highly divergent hybrid individuals (Guo, Ding, et al., 2019; Van Wormhoudt et al., 2011), as well as distinctive and morphologically intermediate shells (Geiger & Owen, 2012). Some haliotid hybrids have also been reported to have paternal mitochondrial transmission (Van Wormhoudt et al., 2011), which can result in heteroplasmy. However, we observed extremely few mitochondrial genome coordinates where there was consistent disagreement between mapped reads that could reflect heteroplasmy (as opposed to DNA damage or sequencing errors). We tentatively retain the current taxonomic treatment in the interests of nomenclatural stability pending additional, ongoing research.

## AUTHOR CONTRIBUTIONS

Kerry Walton, Nicolas J. Rawlence and Hamish G. Spencer conceptualized the study; Kerry Walton performed laboratory work assisted by Alexander J. F. Verry and Lachie Scarsbrook; Kieren J. Mitchell and Lachie Scarsbrook assisted Kerry Walton with data analysis; all authors contributed substantively to the drafts and gave their final approval for publication.

## CONFLICTS OF INTEREST

The authors declare no conflicts of interests.

## Supporting information


Supplementary material

## Data Availability

The initial (near‐complete mitogenomes) and trimmed (as used in phylogeny generation, see Section 2) DNA sequence alignments generated in this study, as well as all raw sequencing data, have been made available at Dryad (Walton et al., 2022; https://doi.org/10.5061/dryad.3ffbg79mq). Consensus mitochondrial genomes are available on GenBank (accession nos ON990036–ON990059).
