# Supplementary material for: Application of palaeogenetic techniques to historic mollusc shells reveals phylogeographic structure in a New Zealand abalone
Source: Mol Ecol Resour. 2022 Aug 21;23(1):118–30. doi: 10.1111/1755-0998.13696 (PMC10087340; doi:10.1111/1755-0998.13696)
Supplement: Supplementary file 1 — Supplementary material [file MEN-23-118-s001.docx]

**Supplemental Information for:**

**Application of palaeogenetic techniques to historic mollusc shell**

**reveals phylogeographic structure in a New Zealand abalone**

Kerry Walton^1^*, Lachie Scarsbrook^1,2^, Kieren J. Mitchell^1^, Alexander J. F. Verry^1,3^, Bruce A. Marshall^4^, Nicolas J. Rawlence^1^ & Hamish G. Spencer^1^

^1^ Otago Palaeogenetics Laboratory, Department of Zoology, University of Otago, PO Box 56, Dunedin 9054, New Zealand

^2^ Palaeogenomics and Bio-Archaeology Research Network, School of Archaeology, 1 South Parks Road, OX1 3TG, University of Oxford, Oxford, United Kingdom

^3^ Centre for Anthropobiology and Genomics of Toulouse, CNRS UMR5288, Université de Toulouse, Toulouse, France

^4^ Museum of New Zealand Te Papa Tongarewa, 169 Tory St, Te Aro, Wellington 6011, New Zealand

*Corresponding author: [walton.kerry@gmail.com](mailto:walton.kerry@gmail.com)

**Table of Contents:**

| **Figure S1** | Pages 2-4 |
| --- | --- |
| **Figure S2** | Page 5 |
| **Figure S3** | Page 6 |
| **Figure S4** | Page 7 |
| **Table S1** | Page 8 |
| **Table S2** | Page 9 |
| **Table S3** | Page 10 |
| **Table S4** | Page 11 |


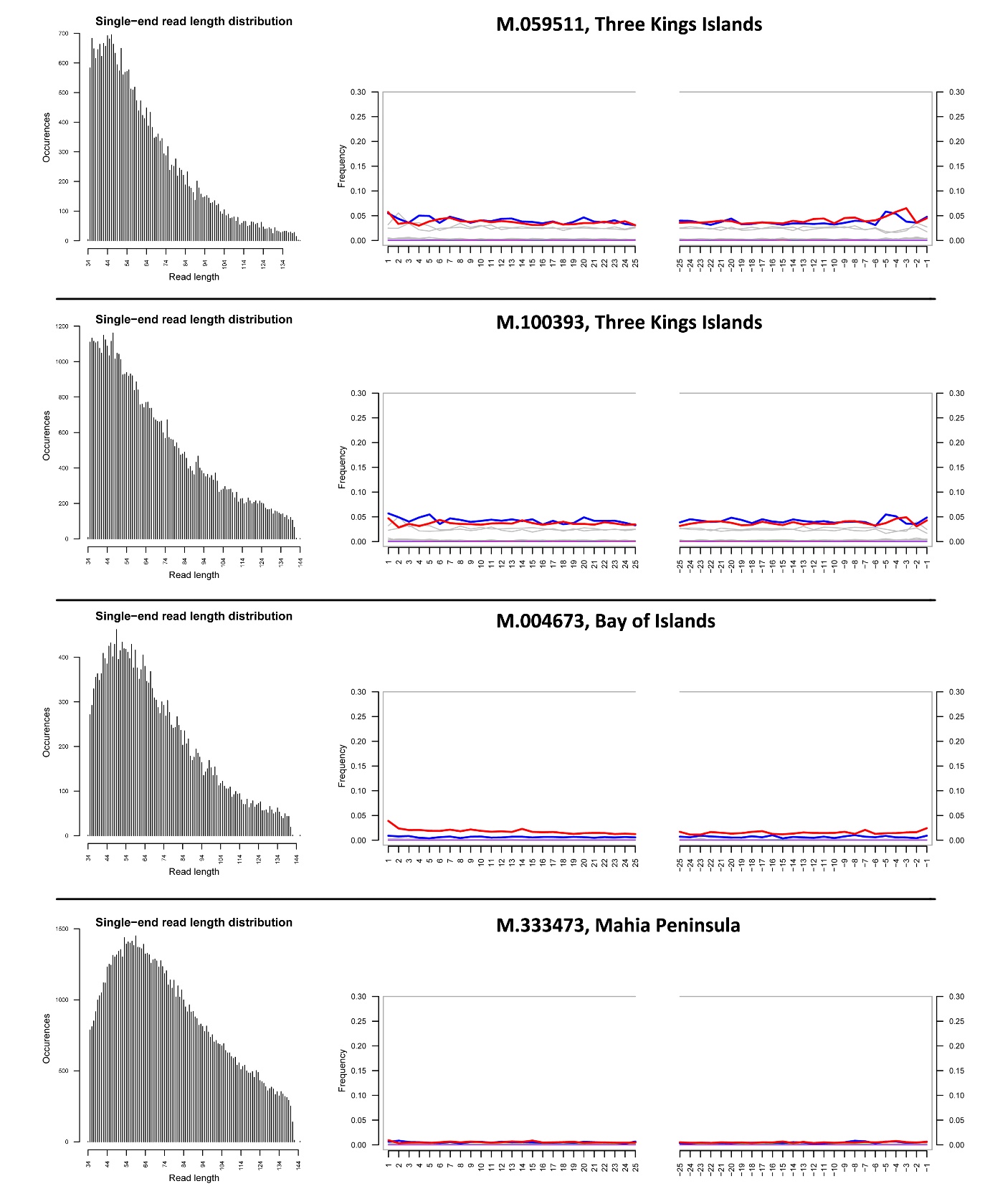
**Figure S1.** DNA degradation information for shell-derived mitogenomes. Histograms of read length (bp) distribution and frequency (left); plots showing proportional accumulation of 5’ C to T (red curve) misincorporations at read termini (center and right).


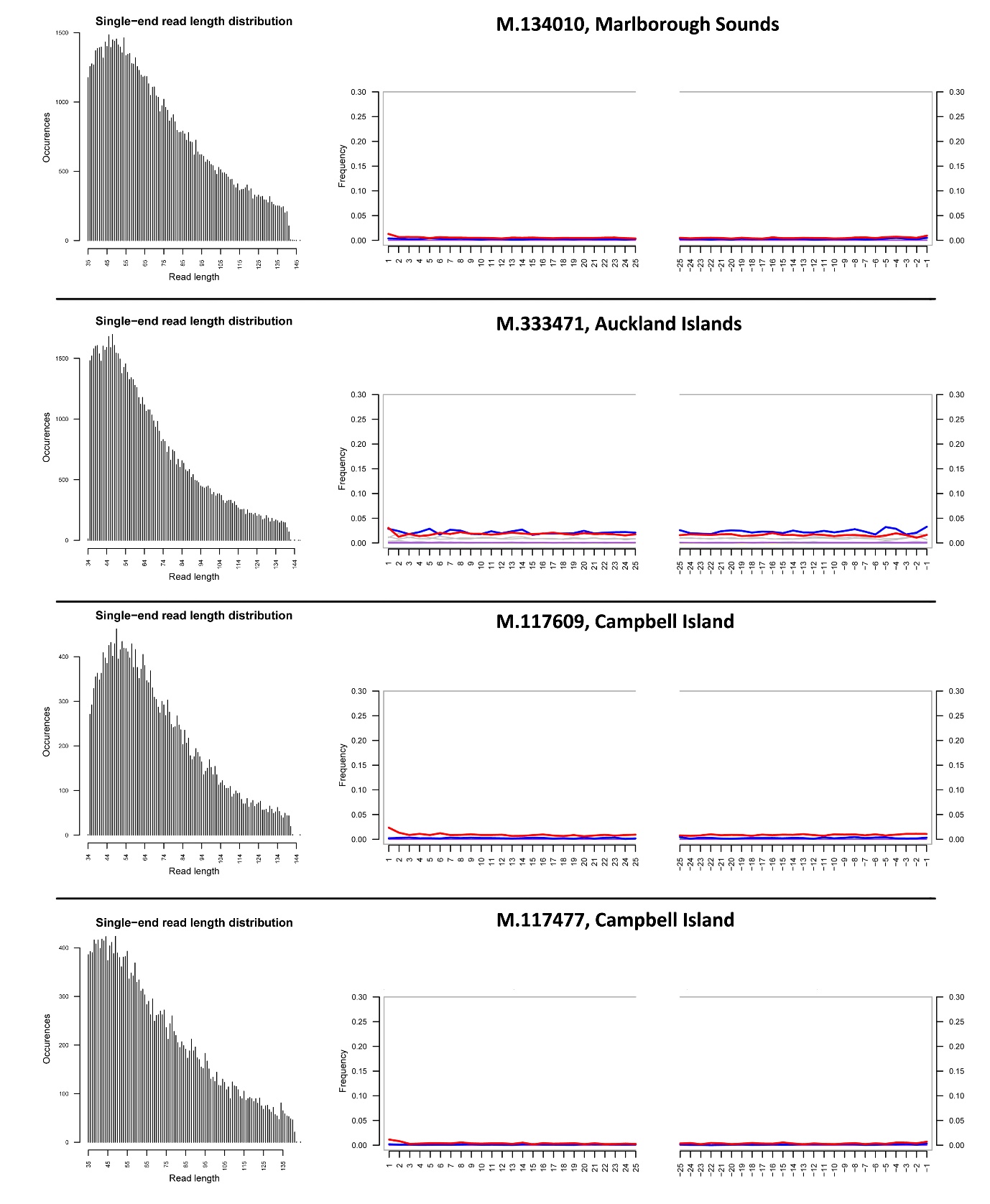
**Figure S1 (continued).** DNA degradation information for shell-derived mitogenomes. Histograms of read length (bp) distribution and frequency (left); plots showing proportional accumulation of 5’ C to T (red curve) misincorporations at read termini (center and right).


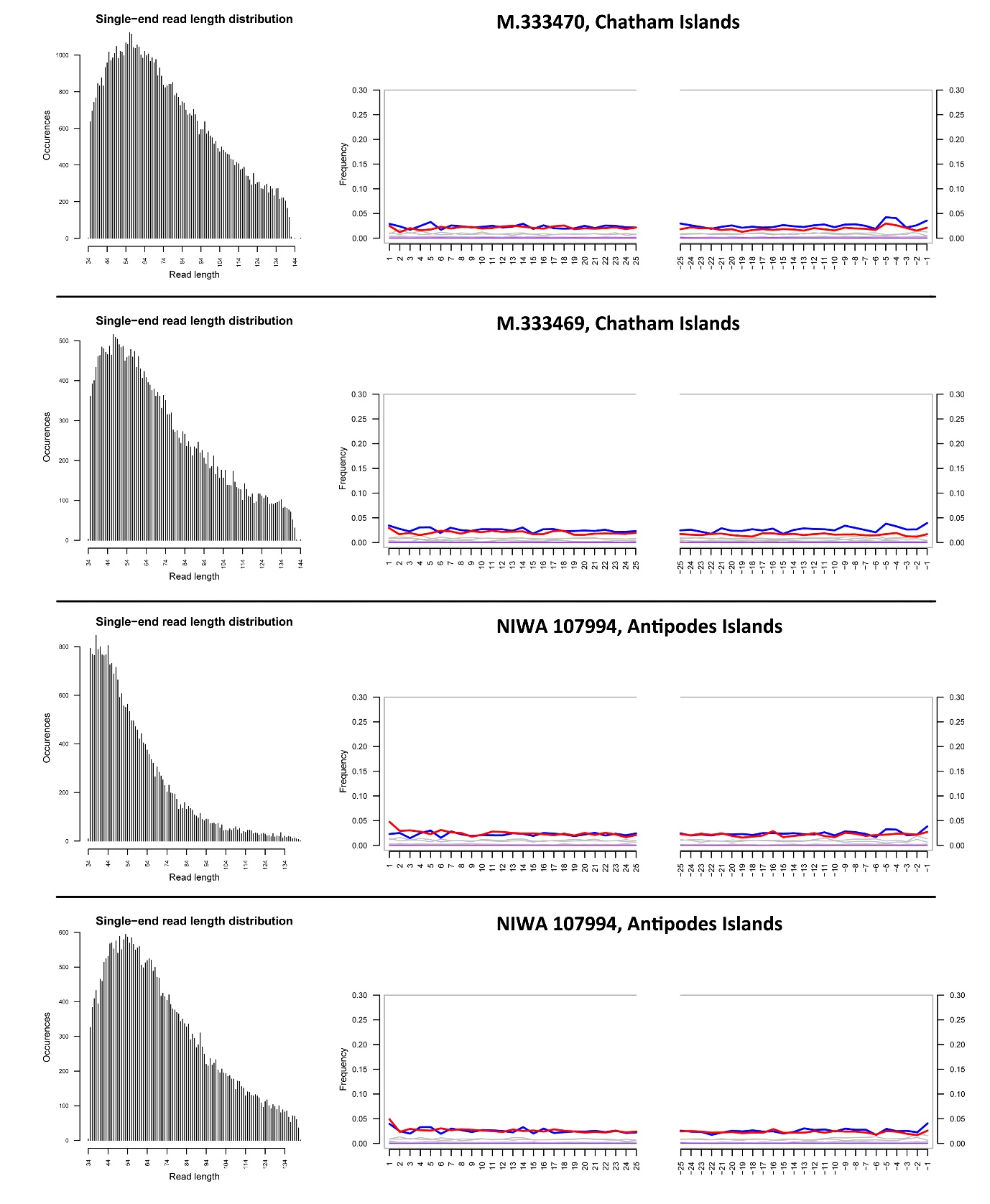
**Figure S1 (continued).** DNA degradation information for shell-derived mitogenomes. Histograms of read length (bp) distribution and frequency (left); plots showing proportional accumulation of 5’ C to T (red curve) misincorporations at read termini (center and right).


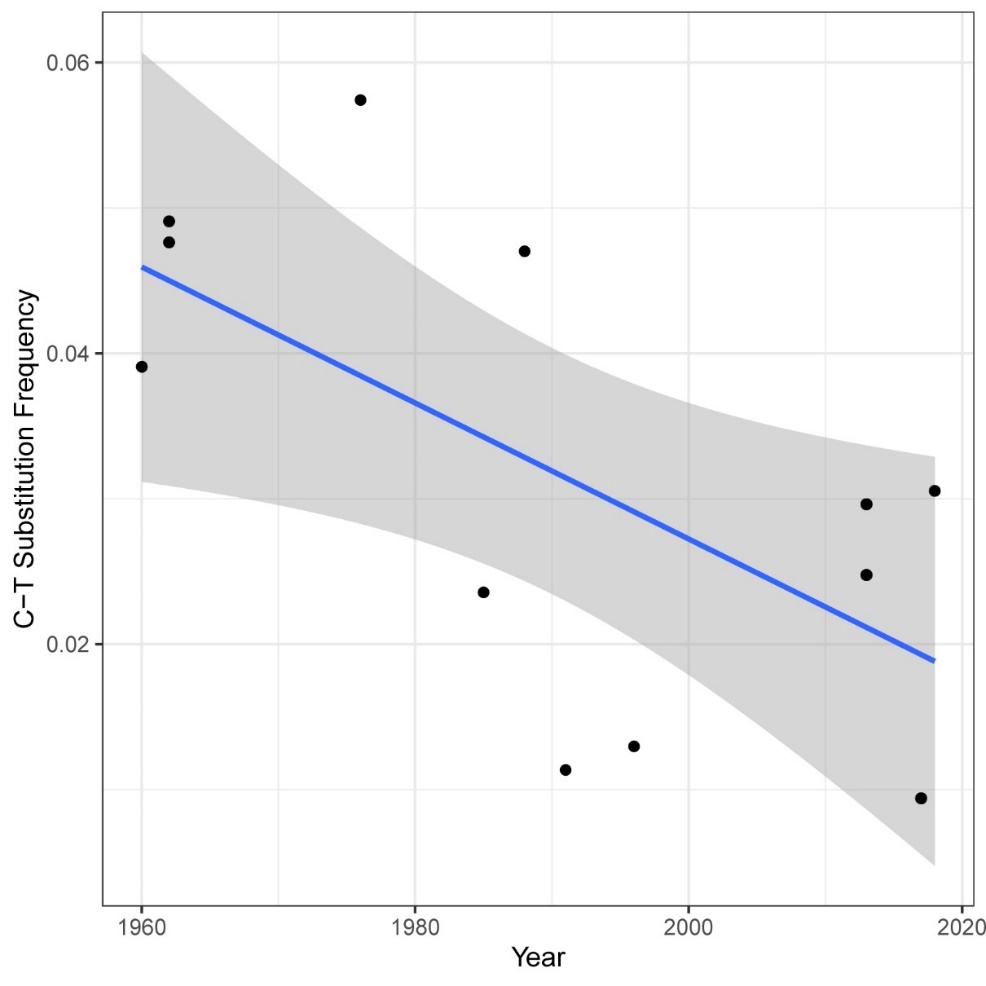
**Figure S2.** Linear regression plot of C-T substitution frequency against year of collection for shell-derived DNA sequences. Grey shading shows variance.


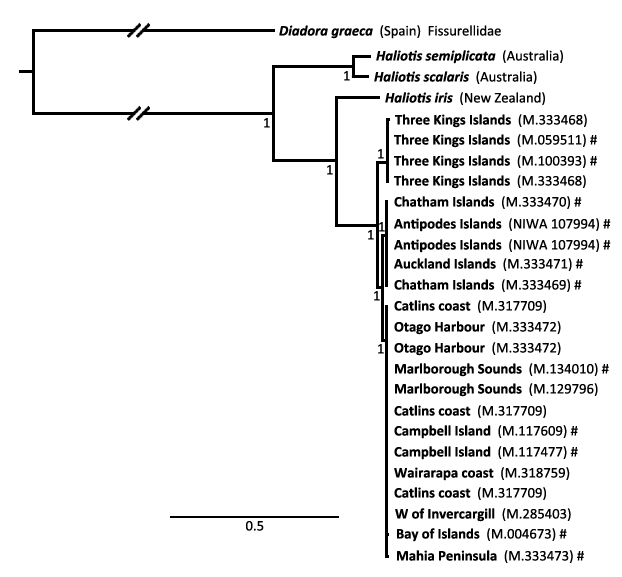
**Figure S3.** Bayesian phylogenetic tree showing relationships in *Haliotis virginea* Gmelin, 1791 generated in Mr Bayes using a 10,059 bp (9,993 bp without gaps for *Haliotis* spp.) concatenated alignment of all mitochondrial protein-coding genes except ATP8 and ND2. Hash symbol denotes sequences sourced from dry-preserved shell.

**
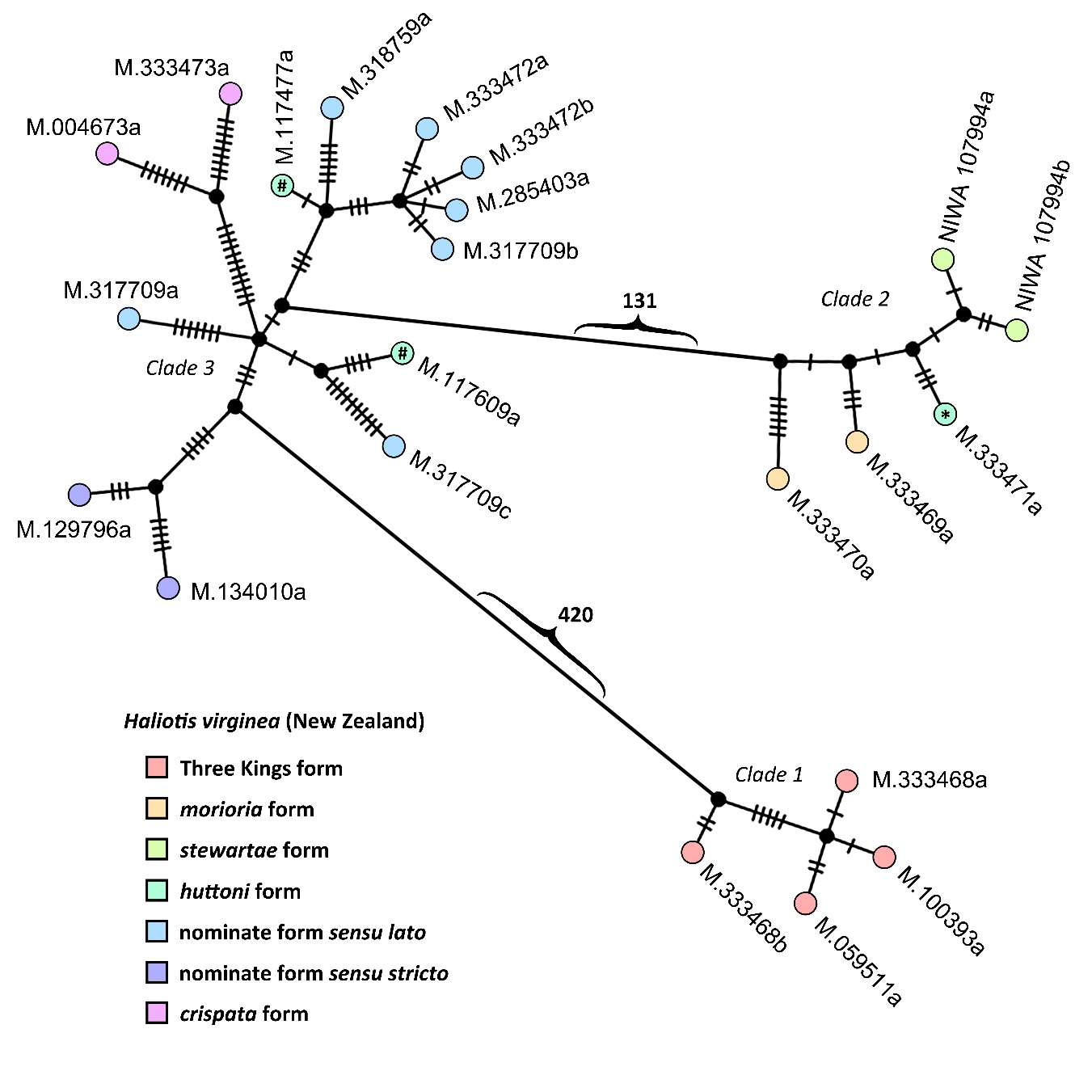
Figure S4.** Haplotype network map (Fig. 3) with annotations to show sequence IDs (corresponding to voucher numbers in Table 1).

**Table S1**. Sequencing information for *Haliotis virginea* Gmelin, 1791 samples. ‘#’ indicates shell-derived sequences; others = tissue-derived.

| **Voucher** | **Mean depth of coverage (standard deviation)** | **Number of unique mapped reads** | **Final sequence length (bp), no gaps** | **GenBank ID** |
| --- | --- | --- | --- | --- |
| M.059511 # | 111.4x (55.1) | 28,182 | 15,916 | ON990040 |
| M.100393 # | 242.5x (129.2) | 56,121 | 15,926 | ON990041 |
| M.333468 (a) | 760.2x (434.8) | 80,848 | 15,921 | ON990038 |
| M.333468 (b) | 846.8x (571.0) | 89,195 | 15,923 | ON990039 |
| M.004673 # | 101.8x (78.9) | 23,252 | 15,904 | ON990057 |
| M.333473 # | 448.4x (197.1) | 93,373 | 15,921 | ON990049 |
| M.318759 | 770.9x (828.8) | 79,216 | 15,922 | ON990055 |
| M.134010 # | 384.3x (188.0) | 84,937 | 15,919 | ON990050 |
| M.129796 | 866.5x (822.6) | 94,538 | 15,926 | ON990051 |
| M.333472 (a) | 803.6x (270.0) | 79,867 | 15,928 | ON990036 |
| M.333472 (b) | 676.6x (283.6) | 71,738 | 15,923 | ON990037 |
| M.317709 (a) | 72.5x (54.9) | 8,190 | 15,919 | ON990052 |
| M.317709 (b) | 213.8x (135.2) | 23,363 | 15,924 | ON990053 |
| M.317709 (c) | 118.6x (138.8) | 12,980 | 15,932 | ON990054 |
| M.285403 | 592.6x (239.0) | 64,274 | 15,923 | ON990056 |
| M.333471 # | 327.2x (147.8) | 77,841 | 15,929 | ON990048 |
| M.117477 # | 98.8x (35.6) | 22,415 | 15,892 | ON990059 |
| M.117609 # | 214.5x (157.5) | 45,150 | 15,887 | ON990058 |
| M.333469 # | 126.9x (81.6) | 28,266 | 15,932 | ON990047 |
| M.333470 # | 333.4x (236.4) | 70,228 | 15,891 | ON990042 |
| NIWA 107994 (a) # | 95.0x (42.0) | 26,110 | 15,917 | ON990043 |
| NIWA 107994 (b) # | 148.6x (96.8) | 33,059 | 15,917 | ON990044 |

**Table S2.** DNA degradation summary statistics for shell-derived mitogenomes.

|  | **Pearson Correlation Coefficient** | **t** | **p-value** | **R-squared (Multiple)** | **R-squared (Adjusted)** | **F-statistic** | **p-value** |
| --- | --- | --- | --- | --- | --- | --- | --- |
| **Mean Fragment Length** | 0.491908 | 1.7867 | 0.1043 | 0.242 | 0.1662 | 3.192 | 0.1043 |
| **5’ C-T Substitution Frequency** | -0.6335375 | -2.5894 | 0.0270 | 0.4014 | 0.3415 | 6.705 | 0.0270 |
| **5’ G-A Substitution Frequency** | -0.09811347 | -0.3118 | 0.7616 | 0.009626 | -0.08941 | 0.0972 | 0.7616 |

**Table S3.** Pairwise sequence similarity (%) values for several haliotid taxa for a 532 bp CO1 alignment trimmed to match the start of the *Haliotis* *rugosa pustulata* Reeve, 1846 sequence (GenBank accession number: AY923918). *H. virginea* Gmelin, 1791 Clade 1 divergence values compared against Clades 2 and 3 (indicated by red arrow) are equivalent to between-species-level divergences in other taxa. Grey indicates >95% sequence similarity.

| **Species (GenBank accession number)** |  |  |  |  |  |  |  |  |  |  |  |
| --- | --- | --- | --- | --- | --- | --- | --- | --- | --- | --- | --- |
| *Haliotis madaka* (AB236716) | 83.6 |  |  |  |  |  |  |  |  |  |  |
| *Haliotis gigantea* (AB236713) | 84 | 98.1 |  |  |  |  |  |  |  |  |  |
| *Haliotis discus*  (AB236701) | 83.8 | 99.4 | 97.9 |  |  |  |  |  |  |  |  |
| *Haliotis rufescens* (NC036928) | 84.6 | 91.4 | 91.4 | 91.5 |  |  |  |  |  |  |  |
| *Haliotis sorenseni* (KP995317) | 84.6 | 91.2 | 91.7 | 91.4 | 97.6 |  |  |  |  |  |  |
| *Haliotis cracherodii* (JF285162) | 82.9 | 91 | 90.2 | 91.2 | 92.5 | 93 |  |  |  |  |  |
| *Haliotis kamtschatkana* (JF285138) | 84.4 | 91.5 | 92.5 | 91.7 | 96.8 | 98.9 | 93 |  |  |  |  |
| *Haliotis walallensis* (JF285131) | 84.6 | 91.2 | 91.5 | 91.4 | 97.6 | 98.7 | 93 | 97.9 |  |  |  |
| *Haliotis* *virginea* Clade 1 (M.333468a) | 87.8 | 83.3 | 84 | 83.5 | 85 | 84.8 | 81.8 | 84.6 | 84.4 |  |  |
| *Haliotis virginea* Clade 2 (M.333469) | 86.7 | 83.5 | 83.8 | 83.6 | 85 | 84.8 | 82 | 84.2 | 84.4 | 95.3 |  |
| *Haliotis virginea* Clade 3 (M.317709a) | 87.4 | 84.2 | 84.6 | 84.4 | 85.9 | 85.7 | 82.9 | 85.2 | 85.3 | 96.1 | 98.9 |
|  | *Haliotis iris* (KU310895) | *Haliotis madaka* | *Haliotis gigantea* | *Haliotis discus* | *Haliotis rufescens* | *Haliotis sorenseni* | *Haliotis cracherodii* | *Haliotis kamtschatkana* | *Haliotis walallensis* | *Haliotis* *virginea* Clade 1 | *Haliotis virginea* Clade 2 |

**Table S4.** Comparison of on-target reads mapped following shotgun sequencing of double-stranded DNA libraries versus enriched single-stranded DNA libraries. seq_collapsed = total number of merged read-pairs; hits_raw = number of merged read–pairs mapped to the *Haliotis iris* mitochondrial genome sequence (KU310895). This analysis was performed using the PALEOMIX v.1.2.9 pipeline; adapter trimming and merging of read-pairs was performed using AdapterRemoval v.2.3.1 (minlength = 30 bp), mapping was performed using BWA v.0.7.15 (aln –n 0.01 –o 2 –l 1024), and results were filtered and sorted using SAMtools v.1.9. Duplicate reads were not removed for this comparison. Results are only reported for the nine specimens that were included in the preliminary shotgun sequencing experiment (data not reported). Note the improvements over the preliminary data reflect a combination of our transition to a single-stranded library approach as well as use of hybridization-capture enrichment.

|  | **Enriched single-stranded libraries** | | | **Shotgun double-stranded libraries** | | | **Enriched vs. Shotgun** |
| --- | --- | --- | --- | --- | --- | --- | --- |
| **Sample** | **seq_collapsed** | **hits_raw** | **% on target** | **seq_collapsed** | **hits_raw** | **% on target** | **x-fold enrichment** |
| M.004673a | 1420684 | 4196 | 0.295% | 602193 | 10 | 0.002% | 177.9 |
| M.333470a | 1193577 | 13847 | 1.160% | 566606 | 64 | 0.011% | 102.7 |
| M.059511a | 1327025 | 9593 | 0.723% | 749082 | 35 | 0.005% | 154.7 |
| M.117609a | 1571086 | 8104 | 0.516% | 684300 | 17 | 0.002% | 207.6 |
| M.134010a | 1403137 | 21367 | 1.523% | 475322 | 18 | 0.004% | 402.1 |
| M.333469c | 1499644 | 5859 | 0.391% | 387364 | 12 | 0.003% | 126.1 |
| M.333471a | 1419832 | 22174 | 1.562% | 536565 | 67 | 0.012% | 125.1 |
| M.333473b | 1507123 | 19402 | 1.287% | 503347 | 37 | 0.007% | 175.1 |
| NIWA 107994a | 1483377 | 12105 | 0.816% | 411799 | 2 | 0.000% | 1680.2 |
| **Mean** |  |  | 0.919% |  |  | 0.005% | 350.2 |
